# Intentional binding reflects pair dynamics and sense of agency in embodied joint action in human-human dyads but not in human-computer dyads

**DOI:** 10.3389/fpsyg.2026.1768740

**Published:** 2026-03-12

**Authors:** Felix Woolford, Keisuke Suzuki

**Affiliations:** Center for Human Nature, Artificial Intelligence, and Neuroscience (CHAIN), Hokkaido University, Sapporo, Japan

**Keywords:** intentional binding, sense of agency, joint action, embodied cognition, human-robot interaction, we-agency

## Abstract

Intentional binding has been proposed as an implicit measure of shared sense of agency in joint action, yet it remains unclear whether it distinguishes between individual and different forms of joint control. We compared intentional binding across individual action, human-computer joint control, and human-human joint control in a physically coupled button-pressing task using haptic feedback devices. Binding magnitude was analyzed both intra-subjectively across conditions and inter-subjectively in relation to reported sense of agency and movement dynamics. Contrary to predictions from the we agency literature, we found no intra-subjective main effect of partner type on binding magnitude. However, within human-human dyads, participants who reported a stronger sense of agency exhibited stronger binding effects, corresponding to emergent leader-follower dynamics in their movement trajectories. No comparable relationship was observed in human-computer interactions. These findings suggest that temporal binding effects primarily reflect sensorimotor predictability rather than intentionality or social context per se. While binding alone does not provide a sufficient marker of “human-like” agency in artificial systems, it may reflect the distribution of predictive control within a dyad and thus serve as a quantifiable signature of how effectively partners co-regulate their actions.

## 1 Introduction

### 1.1 Shared sense of agency and intentional binding

As artificial intelligence systems become increasingly embedded in the physical world, the question of how it feels to interact with them as embodied co-agents becomes increasingly important. There is a growing call for AI to be evaluated not only in domains such as language but also in real-time physical interactions (Zador et al., 2023). This perspective reframes the classic Turing Test in embodied terms, calling for agents that can engage with the world through coordinated, goal-directed movement at a level comparable to living beings. In this context, joint action offers a rich testing ground. When two agents act together, they must adjust to each other's behavior in real time to achieve a shared outcome. In doing so, they engage in a task that is both sensorimotor and social. Crucially, such interactions are not merely about what gets done, but how it feels to do it together. In this study, we consider the idea of “human-like” agency as the extent to which an artificial partner exhibits interaction dynamics that support reciprocal prediction, mutual adaptation, and integrated sensorimotor control comparable to those observed in human-human interaction. On this view, an artificial system may feel more or less “human-like” not because it is attributed beliefs or intentions, but because its behavior affords the same forms of low-level predictive coupling that underlie the experience of fluent joint action.

The sense of agency—the feeling that one is the cause of one's own actions and their effects—offers a lens through which to investigate this experiential dimension (Gallagher, 2000; Frith et al., 2000; Synofzik et al., 2008). Sense of agency can be assessed explicitly through subjective reports of control, or implicitly, through behavioral measures. Intentional Binding (IB) has become a widely used index of implicit sense of agency (Haggard et al., 2002; Haggard and Clark, 2003; Moore et al., 2009). This measure captures the phenomenon of a compression of perceived time between an intentional action and its sensory effect. By asking subjects to estimate temporal intervals under conditions involving intentional actions and various alternative baselines, several studies have suggested that shorter time estimates correlate with a pre-reflective sense of agency over one's actions. Because this temporal attraction is thought to arise from predictive motor processes, it has been proposed as a window into how control is represented at a sensorimotor level. In joint actions, this predictive machinery becomes entangled across individuals: each must anticipate the other's movements and outcomes, blending self-generated and other-generated signals. It has been proposed that shared goals and reciprocal reasoning manifest as a kind of “we-agency”, in which individuals share feelings of intentionality beyond their own individual actions (Tuomela and Miller, 1988; Le Besnerais et al., 2024). In the present study, we use the term we-agency to refer to pre-reflective, sensorimotor forms of shared control and prediction, rather than higher-level social or normative accounts of joint agency, in line with earlier, specifically IB-related studies. Measuring IB in such contexts seems to offer a promising route for assessing the feel of a given joint interaction (Obhi and Hall, 2011b,a; Grynszpan et al., 2019). However, whether IB can serve as a valid marker of felt “jointness” remains an open question. The phenomenon may be robust in individual action but inconsistent in social contexts–sometimes persisting across partners, sometimes attenuating asymmetrically (Weiss et al., 2011; Caspar et al., 2018).

Beyond this, recent study has questioned whether binding is truly driven by intentionality even in the individual case. Temporal attraction between events can arise from multisensory causal integration, even in the absence of voluntary action, and several studies have suggested that prior results are the consequence of mixing such causal factors across conditions, rather than isolating intentionality vs non-intentionality (Desantis et al., 2012; Suzuki et al., 2019; Gutzeit et al., 2023). For example, Suzuki et al. (2019) demonstrated that temporal binding between a keypress and tone could be reproduced when participants passively observed a virtual reality simulacrum of their own arm performing an action, indicating that sensory inference alone can generate the same time compression that the conventional IB hypothesis attributes to motor intentionality. Similarly, Kirsch et al. (2019) and Kong et al. (2024) have shown that binding magnitude scales with the probability of causal linkage rather than with explicit intentionality of action.

In this study, we seek to address the latter challenges by building on research exploring whether intentional binding effects reveal meaningful differences in the sense of agency in human-human joint actions compared to human-computer joint action, in the context of an integrated control task. In doing so, we aim to determine whether IB is sufficiently robust to serve as a quantitative, behavioral measure of how “alive” or responsive an artificial partner feels. If intentional binding fails to capture the nuances of collaboration and mutual adaptation that characterize real joint action, its limits may clarify what future metrics must address–toward richer, embodied indicators of shared intentionality and integrated control.

### 1.2 Intentional binding in joint action

Different conclusions have been drawn regarding how intentional binding interacts with the notion of we-agency. Early research on joint action suggested that the sense of agency could be shared between co-actors. In Obhi and Hall (2011b), pairs performed alternating finger movements that triggered auditory tones, allowing one participant to act as the leader and the other as follower. Both participants exhibited comparable intentional binding, even though followers reported less responsibility for the resulting tones. The authors interpreted this as evidence that both agents experienced a sense of agency over the joint effect, implying a shared model that integrates self- and other-generated actions into a single “we-agency” representation (also Obhi and Hall, 2011a). Later experiments supported this symmetry when both partners maintained mutual predictability. van der Wel (2015) found no difference in binding magnitude between initiators and responders during rhythmic coordination, suggesting that continuous coupling allows both agents to maintain an equally strong implicit sense of control. Similarly, Pfister et al. (2014) showed that participants exhibited IB not only for their own actions but also for their partner's, indicating that shared internal models can extend predictive control to jointly generated outcomes. Together, these studies suggest that as long as both actors retain volitional engagement and reciprocal prediction, a sense of agency is shared across co-actors (Jenkins et al., 2021).

Conversely, other studies have shown that role asymmetries can selectively weaken IB for followers when their individual autonomy is constrained, or their actions are externally triggered. Weiss et al. (2011) and Caspar et al. (2018) reported that individuals executing actions on command showed reduced IB and sensory attenuation. Similarly, Zapparoli et al. (2022) observed in a meta-analysis that followers often report lower explicit and implicit agency during coordinated tasks across multiple experiments. The emerging consensus, therefore, is that IB effects are similar across partners in cooperative, co-regulated interactions where predictive control is preserved for both agents, but followers experience reduced IB when control becomes reactive or hierarchical. However, Grynszpan et al. (2019) did not find a difference between leader and follower dynamics in a shared control task. This may suggest that predictive elements can be retained even by followers if they have continuous sensorimotor feedback.

A comparable tension arises when one member of the dyad is not human but an artificial partner. Human-computer collaborations reproduce many of the same structural asymmetries that undermine follower agency in interactive dyads: the artificial partner is typically deterministic, non-reciprocal, and less sensitive to variance in human actions. Studies using robotic or computer-controlled partners show that binding effects are often weaker for shared or partner-generated outcomes with computer co-actors than in human-human cooperation (Obhi and Hall, 2011b; Grynszpan et al., 2019; Sahaï et al., 2019; Le Besnerais et al., 2025). Giving the artificial partner human-like movement dynamics or traits has not been shown to significantly alter binding effects compared to more overtly robotic partners (Sahaï et al., 2023; Barlas, 2019), although other studies have indicated that presenting an artificial agent as either intentional or non-intentional can affect the feeling of shared agency (Navare et al., 2024).

### 1.3 Joint agency in physically coupled human-computer actions

Most prior studies on we-agency use tasks that involve shared action-initiation roles rather than continuous sensorimotor integration. In such paradigms, partners typically act in pursuit of a common outcome, while retaining independent control over their own physical actions, and coordination is achieved through turn-taking, response inhibition, or strategic timing. For example, Obhi and Hall (2011b) instructed participants to initiate an action unless the partner acted first, and Sahaï et al. (2019) required participants to coordinate responses in a joint Simon task. In these cases, successful coordination primarily relies on higher-level social prediction of the partner's intentions or response tendencies, rather than on continuous mutual adjustment of motor output.

Conversely, in tasks involving integrated sensorimotor control, partners are physically coupled through a shared control interface, such that each agent's movements directly influence the other's ongoing motor behavior. Here, coordination requires continuous adaptation of low-level sensorimotor signals rather than discrete decisions about when or whether to act. In such cases, several studies have shown that participants may struggle to consciously distinguish between human and artificial partners, even when the artificial partner's control policy is simple and yields mechanistic movement trajectories (Reed and Peshkin, 2008; Grynszpan et al., 2019). Nevertheless, in the only study to explore this type of integration in the context of intentional binding, it was found that the human partner in human-computer dyads failed to display markers of we-agency, even when it was assumed that the artificial partner was human. Grynszpan et al. (2019) demonstrated that even though a simple computer-controlled co-actor could mislead subjects into consciously believing that they were interacting with a human (given that they were primed to believe that it was the case), intentional binding effects implicitly suggested that they had a different pre-reflective sense of agency in the interaction compared to human-human interactions. The authors suggest that physical cues in interaction dynamics with human partners induce a sense of we-agency even when there is no conscious recognition of their significance, whereas such interaction dynamics differ in the case of computer-controlled partners. This suggests that mimicking human-like interaction dynamics is crucial for producing convincing replicas of human co-actors, even when subjective reports indicate that the absence of such dynamics is not easily detectable. That study reported stronger intentional binding in both types of joint action than in a no-action baseline, and significantly stronger effects in with-human joint action compared to with-computer joint action, supporting the conventional we-agency hypothesis.

### 1.4 The present study

Given the importance of we-agency as a concept and the diversity of experimental architectures used to study intentional binding in joint action, further study is needed to clarify how binding relates to different forms of control. It is important to note that many prior studies of intentional binding in joint action relied on comparisons with a no-action baseline. However, given that recent study has questioned the interpretability of such baselines (Suzuki et al., 2019; Gutzeit et al., 2023; Kong et al., 2024), no-action baselines may conflate motor engagement with agency-related mechanisms, making them less suitable for isolating the effects of shared control. Therefore, the present study introduces an entirely new architectural basis for the experiment, allowing us to directly compare individual action with human-computer and human-human joint action under matched sensorimotor conditions.

We aimed to clarify the difference between individual actions, human-computer joint actions, and human-human joint actions in terms of IB and sense of agency, and to determine whether such differences manifest in the embodied dynamics of the interaction. In fact, our experiment shows that there is no stronger IB effect in human-human joint action than in either of the other conditions. However, we do identify a clear inter-subjective difference in IB effects, only in human-human joint actions. These results suggest that a temporal binding effect is observable in this form of interaction but does not directly map onto the presence of we-agency, particularly when individual and joint actions are compared under matched sensorimotor conditions.

## 2 Experimental setup

### 2.1 Apparatus

Our experiment adapted the typical interval-estimation paradigm for studying intentional binding, in which participants directly estimate the duration between their action and its effect. This approach is increasingly favored over the original Libet clock paradigm, as it avoids the requirement for subjects to focus on a visual indicator, simplifies task design, and is adaptable to more complex tasks or cognitively engaging settings.

We used a similar experimental setup to Suzuki et al. (2019), adapted for a joint control scenario. To complete each trial, participants had to guide a cursor to one of two virtual buttons and press it, thereby triggering an auditory tone after a fixed delay. They then enter an estimate of the delay between pressing the button and hearing the tone. Cursor control was achieved by manipulating an arm-like input device whose physical position was mapped to a position in virtual space. Control conditions could be either individual or joint. In the individual-control condition, there was a direct mapping between the device and the cursor positions. In joint-control conditions, both participants simultaneously influenced a shared cursor's movement via the mean position of their input devices, requiring continuous coordination to position and activate the button. During training, participants were taught to move the cursor under both conditions. Participants were instructed to press a button for each trial. At the start of each block, they were also asked to try to approximately balance their total number of presses between the left and right buttons. No instruction was given on how to resolve conflicting movements during joint action.

We employed a pair of 3D Systems *Touch* haptic devices to implement this control system (3D Systems, 2025). These devices are motorized, six-degree-of-freedom arms that allow tangible forces to be applied to the user's hand, mimicking the feeling of touching an actual object. While their primary use case is designing and manipulating virtual objects, we have adapted them to work as coupled devices, with the outputs of one device responding to the inputs of the other. Thus, the participants were able to receive natural physical feedback as they pressed the virtual buttons and to feel the effects of discrepancies between their hand movements and the shared cursor movements under the joint-control conditions.

**Figure 1:**
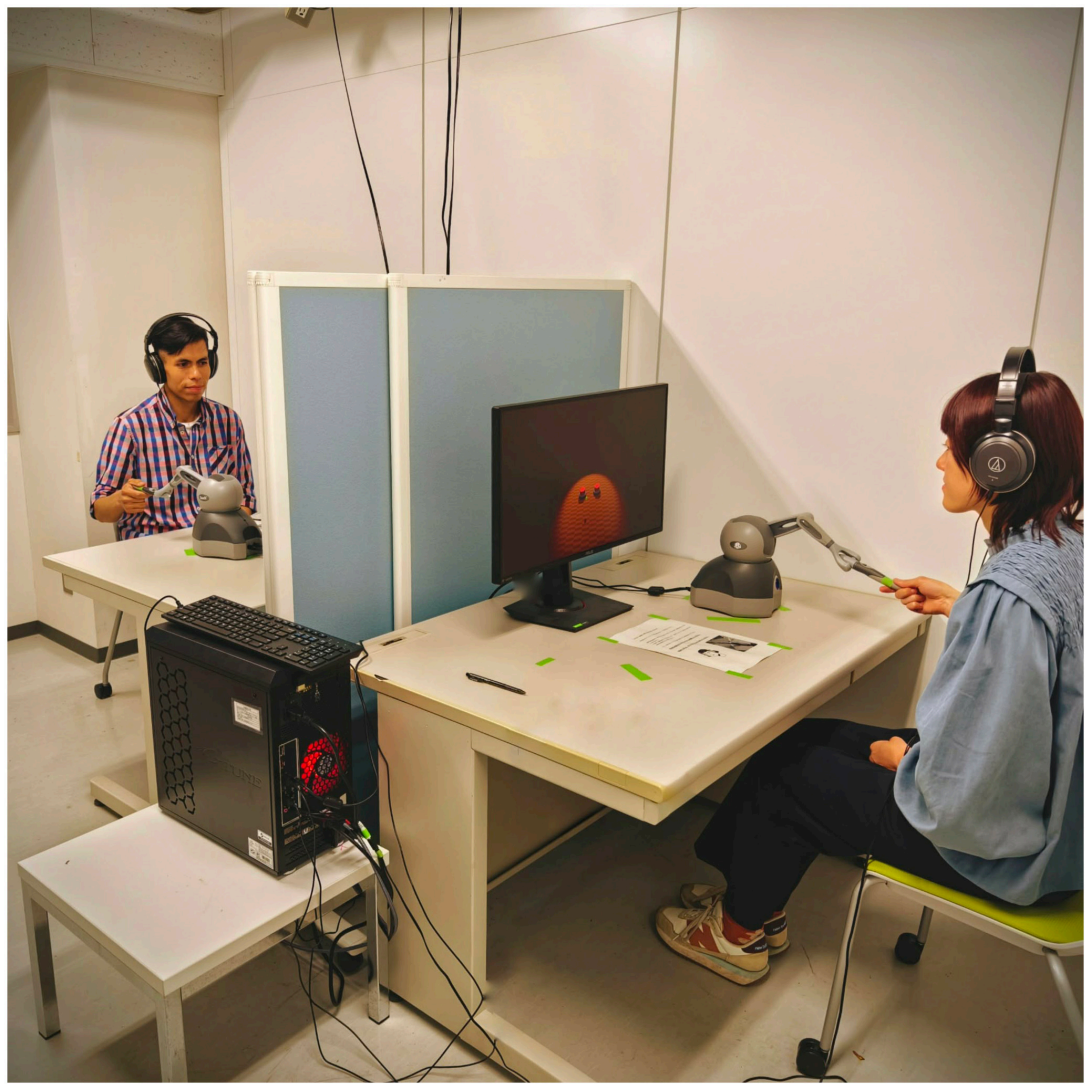
Example of an experiment session in progress. Participant 1 on the right is holding their haptic device and viewing the main display on the monitor. Participant 2 on the left has an identical setup and is hidden behind a screen. All devices are connected to the computer in the foreground.

During an experiment session, two participants were seated at desks facing each other, separated by a dividing screen. Each participant used a 27-inch 144 Hz monitor, headphones, and a *Touch* device located at the side of their dominant hand (Figure 1). To minimize latency, both participants' equipment was connected to a single computer running a single instance of the experiment application. All software was implemented as a custom-designed Unity project using the OpenHaptics Unity plugin (3D Systems Inc., 2022) as the basis for the control system. The application framerate was locked at 144 fps, and the physics timestep was 2 ms. Both the haptic feedback device motors and time-sensitive events were updated in the physics loop to make the device feel smooth and to ensure that real-time delays were accurate to within ±2 ms.

**Figure 2:**
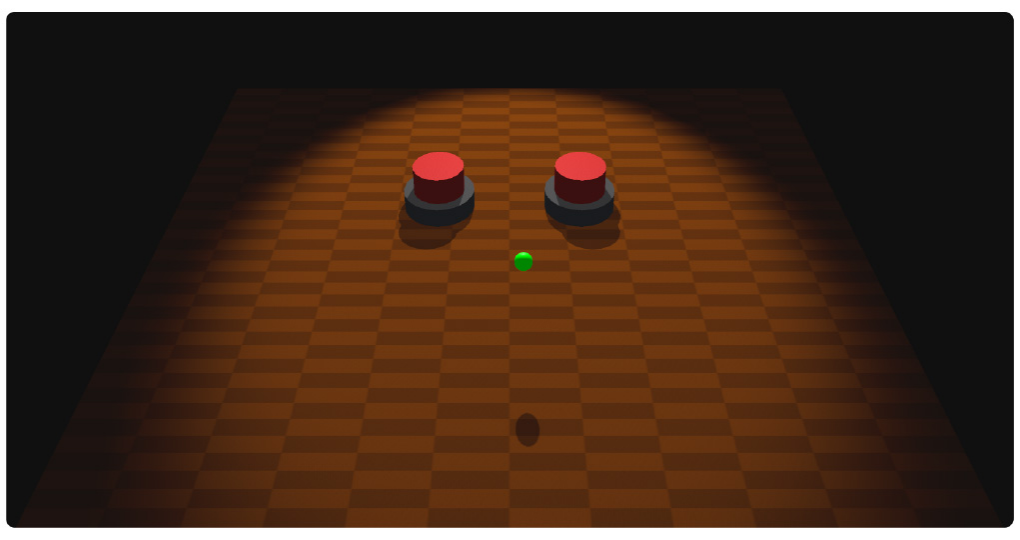
Main view of the experiment display, showing the shared cursor and two pressable buttons.

**Figure 3:**
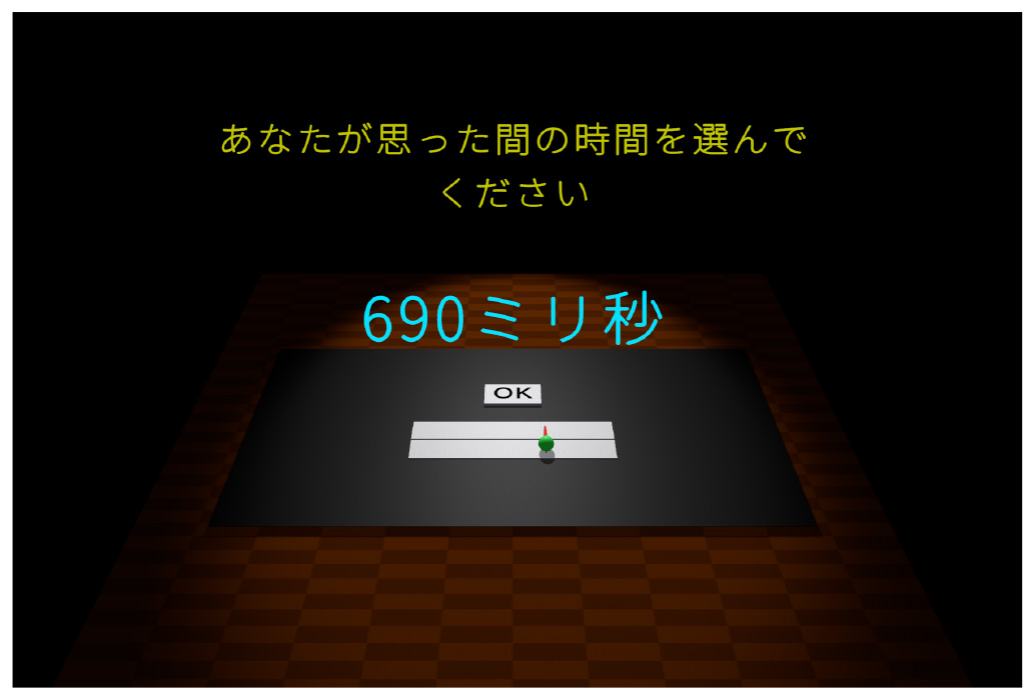
Secondary view of the experiment display, showing an input slider for making time estimates.

The primary view of the experiment consisted of a rendering of a table with two large identical buttons on it to the left and right, and a cursor represented by a small green sphere (Figure 2). The secondary view was the estimate input view, which consists of a horizontal slider and confirmation button, also rendered as virtual objects on a table (Figure 3). All instructions were provided onscreen, in the form of detailed explanations on a black background and short reminder prompts that appeared at the top of the screen while the environment was displayed.

### 2.2 Procedure

The experiment was a 3 × 3 cross-conditional design.

1. Action Condition: (Individual-action (**I**), with-computer-action (**WC**), with-human-action [**WH**)], presented in six blocks of 30 trials with two blocks per condition. As the control conditions were increasingly difficult, blocks were presented in the order **I-WC-WH-I-WC-WH** to maintain participant comfort (through ordering) and control for sequential effects (through repeated cycles). At the start of each block, participants were given on-screen instructions to rest for as long as needed (up to 1 min) and informed of the control condition they would be presented with.
2. Actual Delay: Delays of 300 ms, 500 ms, or 700 ms were inserted between completion of each button press and the sounding of the tone. All three Delay conditions were presented in blocks, with 10 cycles of 3, randomly shuffled at the start of each cycle.

Before the experimental blocks, participants were introduced to using the haptic devices, sharing control of the cursor with the other participant, and estimating time intervals. The training session concluded with 13 individual-action trials, each followed by a message stating the actual delay time during the training program.

To avoid drift in estimate calibration over the course of the experiment, each block after the first was preceded by an additional set of three independent-control trials, after which they were told the actual time delay, identically to the trials in the initial training block.

In the training and recalibration trials, actual delay times were selected from a continuous range between 100 ms and 1,000 ms to obscure the fact that actual delay times in the true trials were limited to 300, 500, or 700 ms.

At the end of the experiment, participants were given a short survey with six questions, each answered on a 7-point Likert scale. The first three questions asked how well they felt they could identify differences between conditions, and the last three asked participants to judge how much agency they felt they had over their actions overall under each condition (see Appendix).

The entire experiment, including training, breaks, and the survey, lasted approximately 1 h.

### 2.3 Control scheme and environmental characteristics

#### 2.3.1 With-human shared control

During a shared control trial, the participants' devices were coupled using a position-position control scheme with a stiff spring coupling (Lawrence, 1993), which gave participants equal control over a shared cursor while they felt a continuous force pulling them toward their partner's position, with a magnitude that scaled with the displacement between individual positions. Essentially, participants would feel little to no resistance in their devices when moving along identical trajectories, with resistance increasing as the trajectories diverged. If one participant did not assert control over their own device, forces were sufficient to pull the other's device, in a dampened reproduction of the other's trajectory. Ultimately, to successfully execute a button press, both participants would have to move their devices in the same direction.

For input, each participant's individual position in virtual space *p*_*virtual*_ was determined by the device pointer's physical position *d* mapped to the virtual space coordinates by a mapping function *f*:


$$\begin{array}{l} p_{virtual_{i}}=f\left(d_{i}\right) \end{array}$$ (1)

For this study, it is sufficient to understand that this function specifies a one-to-one mapping between the Cartesian coordinates of the device's stylus point and those of the virtual environment. The exact function of the device's joint states that determines this mapping is defined in the device's control software (3D Systems Inc., 2018).

The visible shared cursor's position *p*_*cursor*_ was the mean point between the individual positions:


$$\begin{array}{l} p_{cursor}=\frac{p_{virtual_{1}}+p_{virtual_{2}}}{2} \end{array}$$ (2)

For output, the device motors pull the user toward the shared cursor, with a force proportional to the deviation of their individual position from it. The forces on the device are updated at 500 Hz. Thus, at each timestep (δt = 0.002s) the device setpoint *s* is calculated as


$$\begin{array}{l} s_{i}=f^{-1}\left(p_{cursor}\right) \end{array}$$ (3)

and the device motor values are determined via the high-level OpenHaptics Unity plugin HPSpring feature (magnitude = 0.084), which calculates the force on the device at each time step proportional to the difference between the device's position and its setpoint.

#### 2.3.2 Individual control

For individual control, the functions are the same, except that each participant sees an individually owned cursor instead of a shared one, which is fully controlled by them, such that:


$$\begin{array}{l} p_{cursor_{i}}=p_{virtual_{i}} \end{array}$$ (4)

#### 2.3.3 With-computer shared control

For with-computer control, participants each share control of a cursor with a computer partner, which randomly chooses a target button to move toward at the start of each trial and does not alter its target under any condition. As with individual control, each participant sees a separate cursor, but its position is determined as the mean point between their individual position and an algorithmically determined position *p*_*computer*_


$$\begin{array}{l} p_{cursor_{i}}=\frac{p_{virtual_{i}}+p_{computer_{i}}}{2} \end{array}$$ (5)

where *p*_*computer*_ is calculated at each timestep to ensure that *p*_*curso*_*r*__*i*__ follows a direct trajectory from its current location to the target button at a speed slightly faster than average human movement of the device. The effect of this, combined with the haptic feedback, is that the participant is physically pulled through the motion of pressing a button, while still feeling the physical feedback of the environment and maintaining a small amount of control over the precise trajectory of the cursor.

#### 2.3.4 Button pressing

This control scheme allows participants to press either of the two virtual buttons, either jointly or individually, using the shared cursor. The buttons are evenly spaced from the center line on the x-axis, so in real space, they are approximately 20 cm from the start position, either forward, to the left, or to the right. At the start of each trial, participants are guided to position their devices so that the shared cursor appears at a central point directly between the buttons on the *x*-axis and a short distance in front of and above the buttons on the *z*- and *y*-axes. The haptic devices' motors are used to guide them into exactly this position before the coupling force is engaged.

Each button is designed to give the tactile sensation of pressing an actual mechanical button. While the button is being depressed with the shared cursor, an upward force of up to 1.6 N is applied to each device in addition to the previously described coupling forces.

When the button reaches the actuation point, the upward force is removed, and a 20 ms click sound is triggered immediately to provide a realistic tactile and auditory indication of the exact moment the button was activated. Pressing a button triggers a tone (after the conditionally-determined delay), with the left button's tone at 440 Hz and the right button's tone at 523.25 Hz, and a duration of 200 ms (attack = 10 ms, hold = 100 ms, decay 90 ms).

Finally, when the shared cursor contacts the table or button surfaces, additional haptic feedback is applied to each device to simulate the sensation of touching these objects. These forces are calculated using the OpenHaptics Unity plugin's HapticMaterial feature, and are additive with the device coupling forces.

#### 2.3.5 Control in secondary view

The secondary view of the input slider uses the same physical characteristics for feeling the surface of objects, and an individual control system for moving the pointer.

### 2.4 Participants

*N* = 46 Hokkaido University undergraduate students were recruited and took part in the experiment in 23 pairs. Participants were compensated for their time with a 1,500 ¥Amazon gift voucher. The participant number was determined from a power analysis of pilot data with *N* = 12, pre-registered (Woolford and Suzuki, 2025). Five participants' data were excluded from analysis of binding scores based on a preregistered exclusion criterion, in which a one-tailed *t*-test comparing the participant's estimates for 300 ms and 700 ms delays under the **I** failed to reach a relaxed significance threshold (*p*>0.1), indicating they were not reliably tracking actual delay durations. This criterion was designed to identify participants who did not engage meaningfully with the temporal estimation task. Of the excluded participants, 2 participants were partnered with each other, and 3 were partnered with participants whose data were not excluded. Participants selected their own time slots and were paired accordingly. We did not control for the prior relationship between paired participants.

### 2.5 Data collected

Three main forms of data were gathered for each participant: Firstly, time-series data on the trajectories and forces applied to the haptic feedback devices during each trial were sampled at 500 Hz. Secondly, for each trial, a duration judgement in milliseconds of how long the participant perceived the delay between their button press and the onset of the tone. Judgements were collected with an accuracy of 10 ms, but it was assumed that participants would provide only their best guess, and that precision would vary. Finally, at the end of the entire session, three 7-point Likert scale ratings (–3 to +3) were collected for each action condition as a sense-of-agency rating. Additionally, three Likert-scale scores were collected regarding how easily the participant could distinguish each condition and were not used in the analysis.

## 3 Results

**Figure 4:**
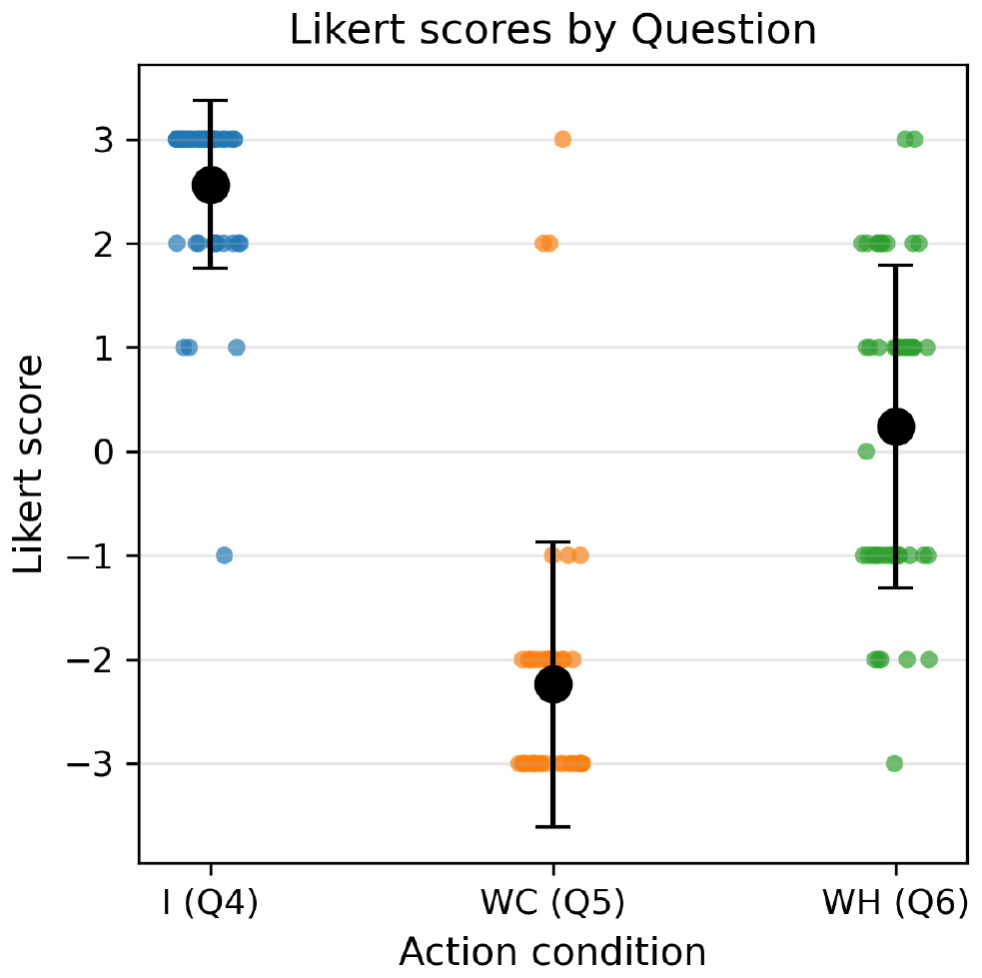
Likert scale responses to questions concerning sense of agency under each action condition. Higher scores indicate a stronger sense of agency over actions. Full questions are presented in Appendix.

Aggregated questionnaire responses for Questions 4, 5, and 6 (presented in Appendix) indicated an expected relationship between action condition and reported sense of agency, with **I** condition (*M* = 2.57, *SD* = 0.81), **WC** condition (*M* = −2.24, *SD* = 1.37), and **WH** condition (*M* = 0.24, *SD* = 1.55). Importantly, and as expected, the distribution of responses for **WH** was bimodal, with participants clearly segregating into positive and negative response groups (Figure 4). Note that positive responses are interpreted to indicate varying degrees of agreement with the statement, negative responses to indicate varying degrees of disagreement, and null responses to indicate no opinion or no confidence in answering the question. These results indicate that, in general, participants experienced a strong sense of agency over individual actions and a weak sense of agency over joint actions in human-computer dyads, while responses to joint actions in human-human dyads were mixed.

### 3.1 Preregistered analysis

We hypothesized (H1) that under the three Action Conditions (**I**, **WC**, **WH**) participants would give higher estimates in **WH** than in **I**, and higher estimates in **WC** than in **WH**. This hypothesis was motivated by the standard interpretation of intentional binding as an implicit marker of sense of agency, leading us to expect that binding magnitude would covary with subjective sense of agency reports across conditions.

Secondly, we hypothesized (H2) that participants who reported feeling they acted intentionally in the **WH** condition would give lower delay estimates for **WH** trials than their **I** estimates, compared with participants who reported not acting intentionally.

These hypotheses would support prior interpretations of IB and we-agency, suggesting that a sense of we-agency does not emerge under the **WC** condition, and that binding effects are weaker for partners who adopt a follower role in a joint action than for those who adopt a leader role.

#### 3.1.1 Intra-subjective binding effects

For H1, we expected to see that average time estimates would reflect differences in intentional binding across action conditions, with the strongest effect in **I** and weakest in **WC**. This hypothesis was not supported by the main effects analysis.

We performed a repeated-measures ANOVA with the action condition (**I**, **WC**, **WH**) and actual delay (300, 500, 700) as within-subjects factors. As expected, there was a significant main effect of the actual delay, *F*(2, 80) = 535.89, *p* < 0.001, Cohen's *f* = 3.66.

Most importantly, and contrary to the hypothesis, the ANOVA revealed no significant main effect of the action condition, *F*(2, 80) = 1.22, *p* = 0.300, Cohen's *f* = 0.17.

Furthermore, a significant interaction between factors was found, *F*(4, 160) = 35.38, *p* < 0.001, Cohen's *f* = 0.94. To further examine the interaction, we conducted three separate 2 × 3 repeated-measures ANOVAs comparing pairs of conditions with Bonferroni correction (α = 0.017). The interaction was significant for all pairwise comparisons: **I** vs **WH**, *F*(2, 80) = 77.24, *p* < 0.001, Cohen's *f* = 1.39; **I** vs. **WC**, *F*(2, 80) = 11.86, *p* < 0.001, Cohen's *f* = 0.54; and **WH** vs. **WC**, *F*(2, 80) = 21.67, *p* < 0.001, Cohen's *f* = 0.74. These results indicate that the pattern of estimates across actual delay intervals differed significantly between all three action conditions.

**Figure 5:**
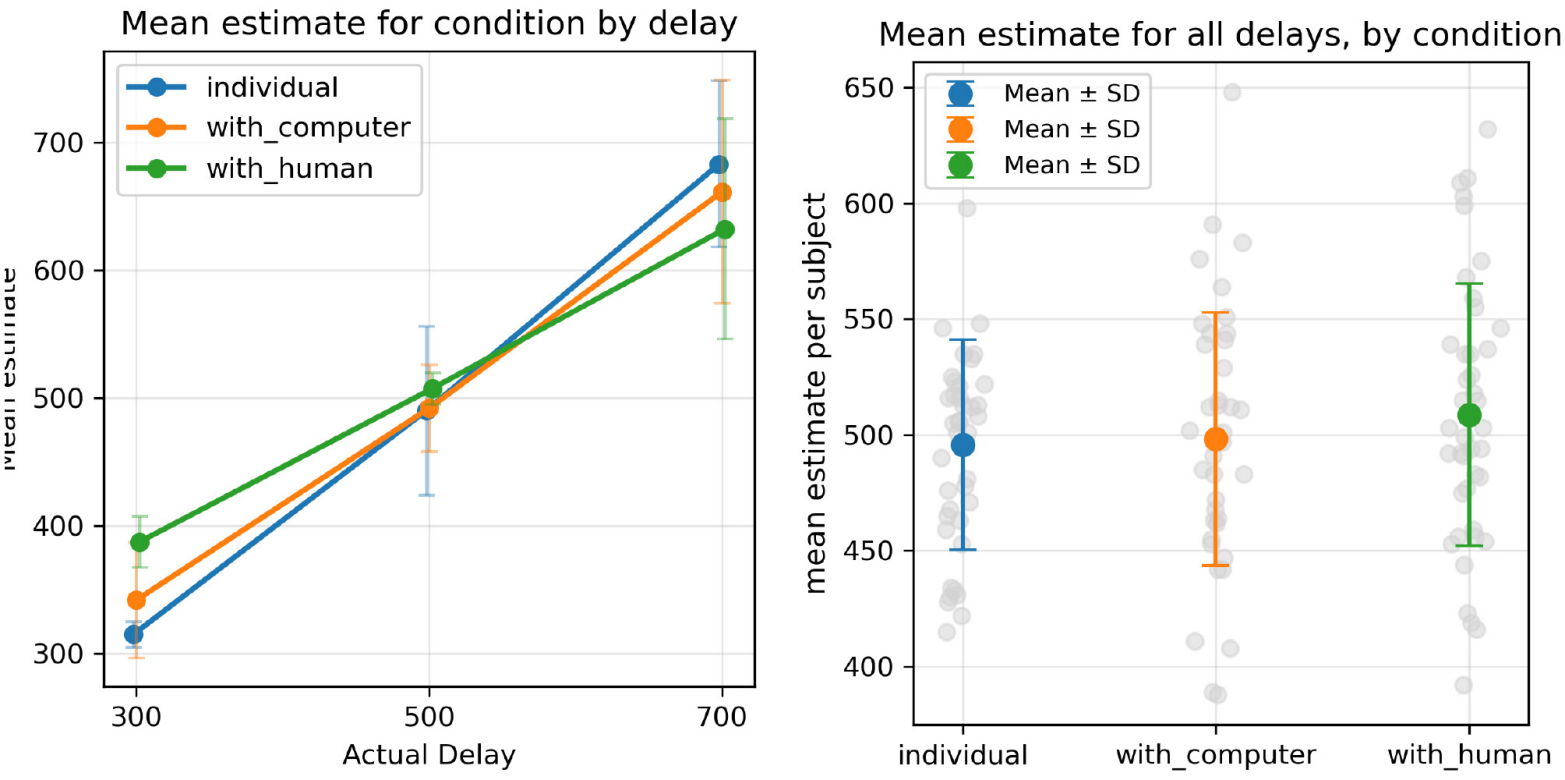
**(Left)** Mean time estimates for all participants, for each action condition and delay condition. **(Right)** Mean estimates for each participant, aggregating all actual delay conditions.

This interaction can be understood by observing the plot in Figure 5. For the individual condition, the grand mean of estimates for each actual delay condition closely maps to the actual delay: *M*_*I*_(300) = 314, *M*_*I*_(500) = 489, *M*_*I*_(700) = 682 with standard deviations *SD*_*I*_(300) = 10.1, *SD*_*I*_(500) = 66.0, *SD*_*I*_(700) = 65.0. In comparison, for the joint action conditions, there appears to be a regression to the overall mean, with higher average estimates for the 300ms actual delay, and lower average estimates for the 700ms actual delay. The effect manifests more strongly in the **WH** condition: *M*_*WC*_(300) = 341, *M*_*WC*_(500) = 491, *M*_*WC*_(700) = 632; *M*_*WH*_(300) = 387, *M*_*WH*_(500) = 507, *M*_*WH*_(700) = 661 with standard deviations *SD*_*W*_*C*(300) = 45.2, *SD*_*W*_*C*(500) = 34.0, *SD*_*W*_*C*(700) = 87.5, *SD*_*W*_*H*(300) = 19.9, *SD*_*W*_*H*(500) = 12.2, *SD*_*W*_*H*(700) = 86.3.

#### 3.1.2 Inter-subjective binding effects

For H2, we hypothesized that participants would show different binding effects in the **WH** condition depending on how strongly they felt a sense of agency over the joint action in that condition. This hypothesis was supported by a strong effect observed in the results.

Participants were grouped according to whether they gave a positive (high-SoA group) or negative response (low-SoA group) on the Likert scale to the statement, “In trials where the other person was controlling the pointer jointly with me, I felt like I was intentionally pressing the button” (statement 6). Participants who responded positively were taken to have generally felt a stronger sense of agency over the resultant actions, and participants who responded negatively were taken to have felt a weaker sense of agency over the actions. Of the 46 participants, 1 provided a neutral response to this question; however, that participant was excluded from this analysis due to the preregistered exclusion criterion. Of the 41 participants who were included, 22 were in the high-SoA group, and 19 were in the low-SoA group.

**Figure 6:**
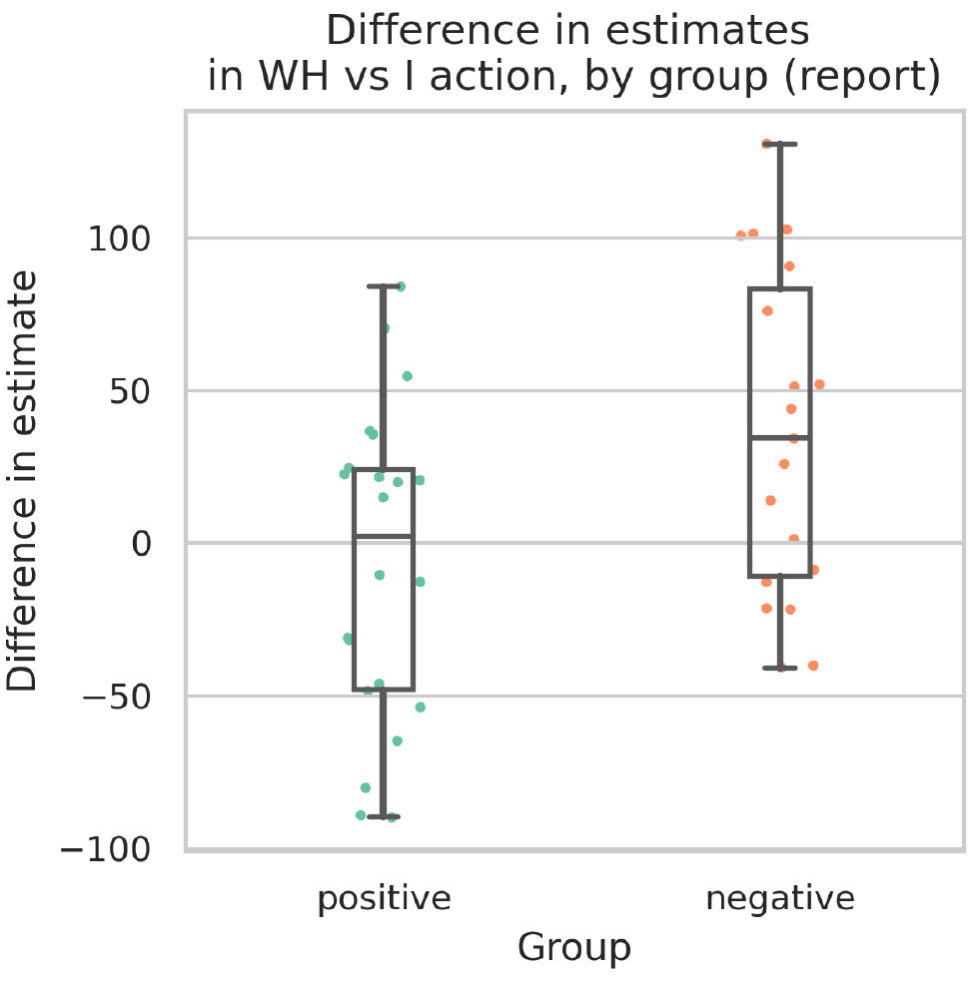
Comparison between time estimates in **I** condition vs **WH** condition for each participant, grouped by reported sense of agency. A positive value indicates that time estimates are longer under **WH** for that participant (i.e., indicating a weaker binding effect).

**Figure 7:**
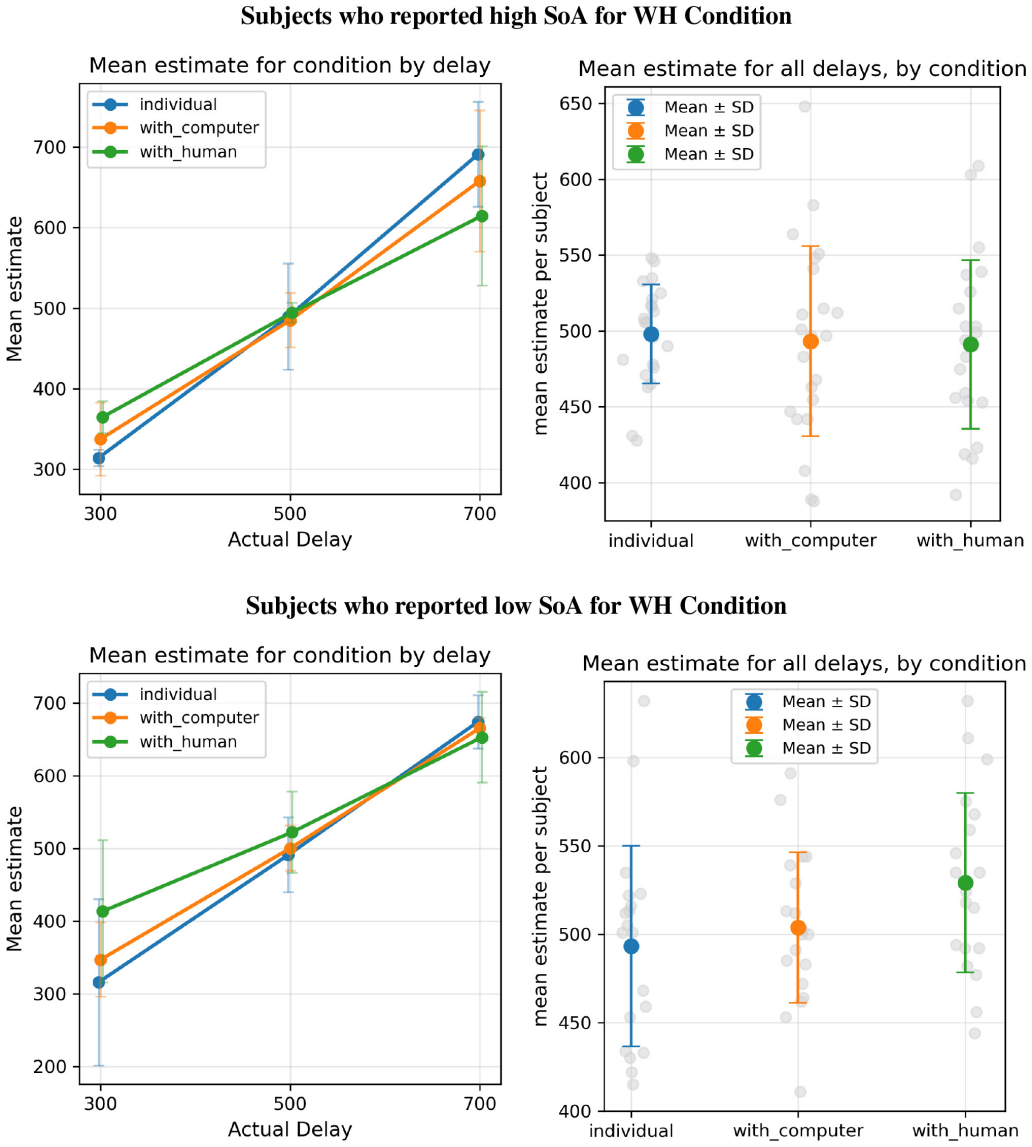
The same mean estimate plotting as in Figure 5, but with participants split by reported SoA grouping as in Figure 6.

For each participant, their average estimate for the **I** condition was subtracted from their average estimate for the **WH** condition, yielding an index of the relative binding effects between the two conditions. These indices were then grouped according to the participants' questionnaire responses. A one-tailed independent-samples Welch's *t*-test showed that participants in the high-SoA group had significantly stronger IB scores than in the low-SoA group, *t*(37.63) = –2.60, *p* = 0.0067, Cohen's *d* = 0.82 (Figure 6). Figure 7 also illustrates how the higher estimates for the low-SoA group are consistent across actual delay conditions.

As a *post-hoc* verification that this group difference was not driven by delay-specific effects, we additionally conducted a mixed ANOVA with SoA group (high vs. low) as a between-subject factor and delay condition as a within-subject factor. This analysis revealed a medium-to-large main effect of SoA group (Cohen's *f* = 0.36), consistent with the preregistered Welch's *t*-test. There was also an expected strong main effect of delay condition (Cohen's *f* = 2.16). The interaction between the SoA group and delay condition was small (Cohen's *f* = 0.09), indicating that the difference in binding strength between groups was consistent across delay conditions.

### 3.2 Exploratory analysis

#### 3.2.1 Exploratory analysis of estimate variability

As an exploratory control analysis, we examined whether within-participant variability in time estimates varied across action conditions and delays. For each participant, we computed the standard deviation of time estimates separately for each action and delay condition. These SDs were entered into a mixed ANOVA with action condition and delay as within-subject factors.

This analysis revealed no significant main effect of Action Condition, *F*(2, 80) = 1.93, *p* = 0.15, *f* = 0.22, and a strong expected main effect of Actual Delay, *F*(2, 80) = 50.85, *p* < 0.001, *f* = 1.13. Crucially, there was a significant Action Condition × Actual Delay interaction, *F*(4, 160) = 7.20, *p* < 0.001, *f* = 0.42, indicating that differences in estimate variability across action conditions varied with delay.

#### 3.2.2 Correlation between Leader/Follower dynamics and reported SoA and IB

Given that the questionnaire was coarse, asking only for a general impression of each condition after the entire experiment was complete, we conducted further exploratory analysis to assess how well those impressions correlated with leader-follower pair dynamics as reflected in the movement trajectories.

To assess leader-follower dynamics from participants' movement trajectories, we used two independent metrics for each pair. For the first metric, we computed a threshold-crossing analysis (TC), in which each pair was assigned a score as the proportion of trials in which Partner 1 first crossed a predetermined movement threshold toward the final position (He and Yuan, 2001). The threshold was defined as the smallest deviation from the center along the x-axis for which the side of the final position could be determined with probability *p*>0.99, based on the distribution of final positions across all trials for all participants under the **WH** condition.

Each pair's overall score was computed as the mean leadership score of Partner 1 across all trials.

For the second metric, we applied dynamic time warping (DTW) to each pair of movement trajectories to determine an optimal non-linear temporal alignment between the two time series. DTW is an algorithm that warps the time axes of two sequences to minimize the cumulative distance between matched points, allowing one trajectory to be stretched or compressed in time relative to the other while preserving the temporal order of events (Stübinger and Walter, 2022). From the resulting warping path, we extracted a lead-lag summary: a value of 0.5 indicates an even balance of leading and lagging behavior, values approaching 1 indicate that Partner 1's trajectory consistently led Partner 2's across the aligned path, and values approaching 0 indicate the reverse. This approach quantifies how one partner's movement tended to precede or follow the other's.

Because both metrics yield values between 0 and 1, indicating relative dominance between partners, scores were transformed so that values always reflected the degree of dominance of the more dominant partner within a dyad. Specifically, if a raw score *s* < 0.5 indicated that Partner 2 was more dominant than Partner 1, the score was adjusted as *s*_adjusted_ = 1−*s*. After this transformation, values closer to 1.0 indicated strong dominance by one partner, whereas values near 0.5 indicated a more balanced interaction. Although both metrics quantify leader-follower asymmetries, they capture different aspects of coordination: the TC metric indicates which partner tended to initiate actions, whereas the DTW metric reflects relative commitment across the entire movement trajectory.

To relate leader-follower dynamics to reported sense of agency (SoA), correlation analyses were conducted for each metric. For each dyad, we classified outcomes as *correlated* when the partner identified as more dominant by the metric also reported higher SoA on the questionnaire, and as *anticorrelated* when the less dominant partner reported higher SoA. Dyads in which neither or both partners reported higher SoA were considered uninformative and excluded from this binary analysis.

**Figure 8:**
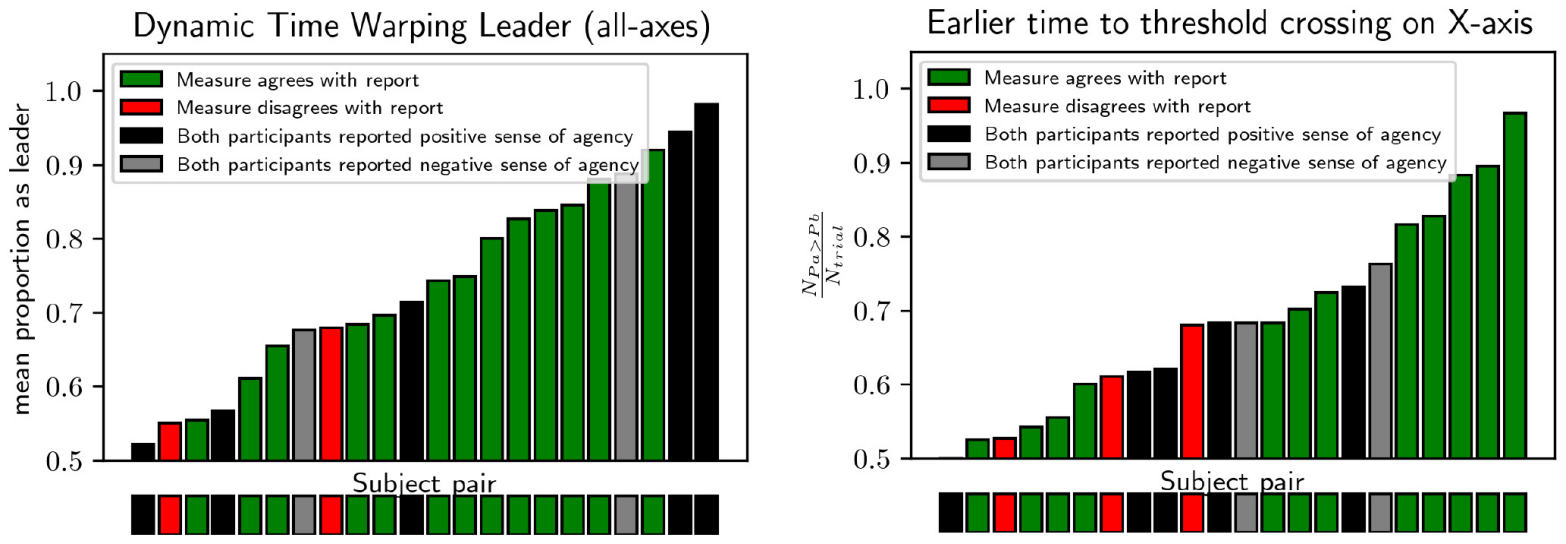
Leader-follower scores for each pair of participants. Values close to 1 indicate that one partner was always the leader according to the given metric, values close to 0.5 indicate that roles were equally mixed across trials. Colors indicate whether the partner with the higher leader score was also in the high-SoA group, as determined from questionnaire responses.

As illustrated in Figure 8, leader-follower dynamics were positively related to reported SoA across **WH** joint trials. For both metrics, correlated outcomes occurred significantly more often than expected by chance, with *p* = 0.0176 for the TC metric and *p* = 0.0037 for the DTW metric. Additional distributional comparisons showed consistent trends in the expected direction but did not reach significance (Mann–Whitney *U* tests: *p* = 0.1165 for TC and *p* = 0.0571 for DTW), likely due to the small number of anticorrelated cases (2–3 out of 15 informative dyads). Despite this limitation, the convergence across analyses supports a positive association between leader-follower asymmetry and SoA reports.

**Figure 9:**
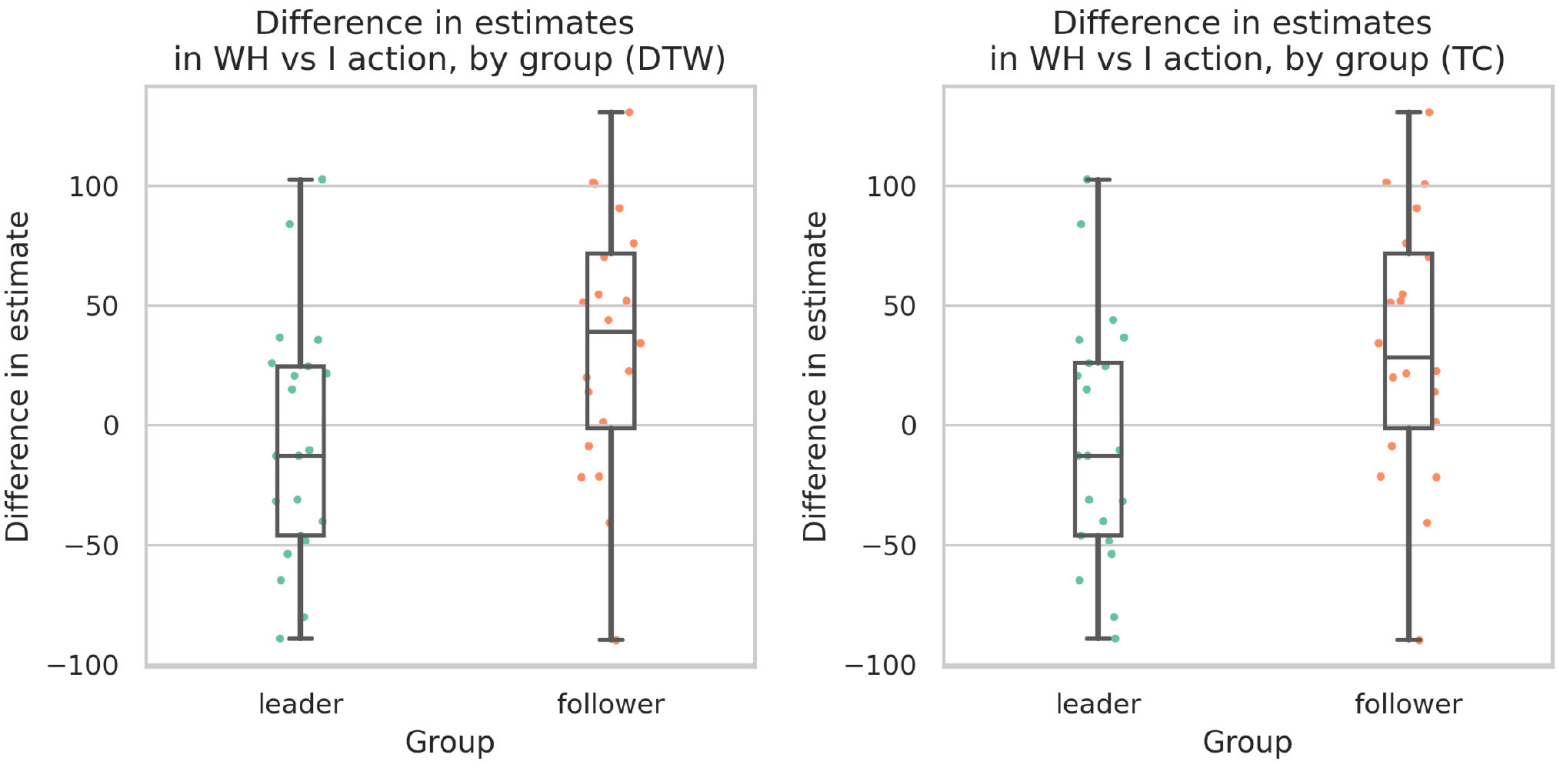
Same plotting as in Figure 6 but with groups split according to leader-follower scores instead of by reported SoA.

Given this relationship, participants were subsequently grouped as leaders or followers based on whether they had the higher leadership score in their dyad. These groupings were defined to provide a comparative parallel to the grouping by reported SoA. Then, inter-subjective intentional binding (IB) effects were compared between groups (Figure 9). When groups were defined using the TC metric, leaders showed significantly stronger IB than followers (one-tailed independent-samples Welch's *t*-test: *t*(38.57) = −2.36, *p* = 0.011, Cohen's *d* = 0.74). The same pattern was observed when grouping was based on the DTW metric (*t*(38.42) = −2.52, *p* = 0.008, Cohen's *d* = 0.79). Although DTW-based groupings showed slightly stronger effects than TC-based groupings, and the reported SoA showed a slightly stronger effect than both, further research would be required to establish reliable differences among the metrics.

#### 3.2.3 Analysis of human-computer dyad dynamics and IB

Using the DTW metric to evaluate leader-follower dynamics in the **WC** action condition showed that the computer-controlled partner consistently took a leader-like role in the dyad, as expected given the control policy. This correlated clearly with the reported sense of agency for that condition, with all but two participants responding negatively on the Likert scale.

Given the lack of distinct groupings for the **WC** condition through either reported SoA or implicit leader-follower dynamics, it was not possible to analyze inter-subjective IB effects in the same way as for the **WH** condition. However, to determine whether the observed IB differences between high-SoA and low-SoA groups remained even under different conditions, we also used the same grouping (reported SoA for **WH**) to evaluate differences in IB effect under **WC**. A one-tailed independent-samples Welch's *t*-test showed *t*(38.91) = –0.85, *p* = 0.200, Cohen's *d* = 0.263, indicating that there was no significant difference between groups. This supports the interpretation that there is a correlation between low reported SoA and weaker IB under the **WH** condition, as opposed to there being a tendency for participants in the low-SoA group to report higher estimates regardless of action condition.

## 4 Discussion

The study investigated how intentional binding and the subjective sense of agency vary across individual, human-computer, and human-human joint actions, and how these experiences relate to emergent leader-follower dynamics in the movements. The preregistered analyses did not show a main effect of action condition on binding, but they did reveal systematic inter-individual differences: participants who reported stronger agency in the human-human condition also exhibited a stronger temporal binding effect. *Post-hoc* exploratory analyses of trajectory data further indicated that human-human dyads tended to develop a dynamic in which one partner assumed a leader role significantly more often than expected by chance. This inference follows from comparing the threshold-crossing (TC) results to a null model in which neither participant consistently adopts a leader role, such that each partner would be expected to initiate actions with probability *p* = 0.5 on each trial. Under this model, with 60 trials per dyad, only 5% of dyads would be expected to exhibit adjusted TC scores greater than or equal to 0.606. Conversely, 17 out of 22 dyads exceeded this value, indicating that stable leader-follower asymmetries occurred substantially more often than predicted by random alternation. Moreover, these asymmetries were associated with higher reported agency among leaders and lower reported agency among followers. These dyadic dynamics were also correlated with differences in intentional binding effects. However, no comparable relationships among reported sense of agency, pair dynamics, and intentional binding were observed in the human-computer condition.

### 4.1 No difference in population-wide average time estimates across action conditions

The absence of significant differences in intentional binding across the three action conditions challenges the common assumption that a partner's rigidity or artificiality necessarily weakens the sense of agency. Prior studies have often interpreted reduced binding with non-human or computer partners as evidence that social or intentional features–or at least an imitation of these–are necessary for “we-agency” to emerge (Sahaï et al., 2019, 2023; Obhi and Hall, 2011a; Grynszpan et al., 2019). However, the present results suggest that temporal estimations do not systematically differ in ways expected if binding were reduced, even when the partner's contribution is highly rigid, provided that the causal relation between action and outcome remains predictable.

Rather than proving an emergence of we-agency under the with-computer condition, however, the results are more consistent with the view that temporal binding reflects sensorimotor causal integration rather than a specific sense of intentionality *per se* (Suzuki et al., 2019; Gutzeit et al., 2023; Kong et al., 2024). All three conditions involved consistent relationships between sensorimotor dynamics and causal effects. In other words, the arm movements and sensations of button pressing were consistent regardless of whether the action was individual or joint, and whether it was intentionally initiated or forced.

Therefore, the consistency of mean estimates suggests that introducing a partner, artificial or otherwise, does not inherently change the mechanisms producing a temporal binding effect. What matters is whether the individual can predict how their own actions, in combination with the partner's actions, contribute to the outcome. Specifically, this predictive capacity depends on the stability of the underlying sensorimotor dynamics, rather than on whether the partner is an intentional agent or an artificial system.

Notably, in the present design, participants were explicitly informed of the action condition at the start of each block. While this approach reduced variability in how quickly participants inferred the interaction structure, it may also have reduced social attribution effects compared to studies in which partner identity or intentionality was ambiguous. Future research could systematically vary both partner predictability and the explicitness of partner information to further dissociate these influences.

### 4.2 Regression to mean in joint action condition estimates

Despite the absence of a significant main effect, the significant interaction effect between the factors indicates that there is, nevertheless, a relationship between action conditions and time estimates. This is observed in the tendency toward middle-range estimates in the **WC** and **WH** conditions compared to the **I** condition (Figure 5).

A similar interaction between action condition and delay was also reported by Grynszpan et al. (2019), although the range of actual delay times differed substantially between studies, with ranges of 300–700 ms in our study compared to 700–1,300 ms in the earlier study. The presence of a comparable interaction structure across both studies, despite different actual delay ranges, suggests that the effect is unlikely to be driven solely by specific delay durations (i.e., it is not that a more pronounced binding effect occurs at longer delay times). This also makes it unlikely that the interaction reflects a simple non-linear binding effect restricted to a particular delay range, or floor/ceiling effects tied to extreme intervals. Rather, participants seemed to experience a diminished ability to distinguish between different actual time intervals under the **WC** and **WH** conditions. In other words, the regression to the mean likely reflects decreased accuracy and confidence in estimations under the **WC** and **WH** conditions compared to the **I** condition, rather than a linear compression of perceived time under different action conditions. Consistent with this interpretation, the exploratory analysis of within-participant variability revealed that differences in estimate variability across delay conditions interacted with action conditions. This may reflect processes related to, but distinct from, classical IB effects. Rolke and Hofmann (2007) showed that temporal uncertainty can undermine perceptual discrimination, and under classical interpretations, increased temporal unpredictability reduces intentional binding effects. Compared to the individual actions in our study, the joint-action context may introduce temporal ambiguity about the timing of the action itself, leading to increased variability in temporal judgements rather than linear temporal compression. This is consistent with interpretations that link the strength of sense of agency to the precision of sensorimotor predictions (Synofzik et al., 2013). We do not exclude the possibility that multiple factors contribute to this pattern, such as general increases in estimation error at longer intervals. However, the fact that variability and regression effects interact with action condition argues against a purely time-based floor or ceiling explanation. It may be that the regression toward the mean estimates indicate that participants are genuinely experiencing altered temporal perception in joint-action conditions, but manifesting as reduced precision rather than uniform temporal attraction. Such an effect could not be detected by the conventional approach to measuring binding effects, which compares mean estimates across conditions.

### 4.3 Reported sense of agency correlates with temporal binding in human-human joint actions

Although intra-subjective comparisons across conditions failed to show compelling evidence of conventional IB effects, inter-subject comparisons revealed that the dynamics of partnership roles correlated with a clear effect on intentional binding, which was obscured by population-wide analysis. Participants who reported intentionally pressing the button in the **WH** condition showed significantly shorter time estimates than those who disagreed. Importantly, this relationship does not contradict the view that temporal binding primarily reflects sensorimotor causal integration. Rather, it suggests that individual differences in experienced agency may covary with the extent to which participants integrated the joint sensorimotor contingencies underlying the action. Consistent with this interpretation, the effect was stable across actual delay conditions (Figure 7), indicating a genuine temporal compression effect rather than a regression-to-the-mean artifact. The presence of measurable inter-subjective IB effects is consistent with findings by Imaizumi and Tanno (2019), who similarly reported that individual differences in experienced agency predict the magnitude of temporal binding. Our experiment suggests that the observations reported by Imaizumi and Tanno (2019) can be understood as consistent with views in which intentionality *per se* is not the primary causal driver of temporal binding.

The exploratory analysis of leader-follower dynamics strengthens this interpretation. The spontaneous emergence of partners adopting leader and follower roles is consistent with other studies in joint action (Kropivšek Leskovar et al., 2021). Across dyads, partners who initiated or temporally led the joint movement more frequently also reported stronger agency over the shared actions. These correlations held for both threshold-crossing and DTW-based measures of leadership, and the predominance of correlated over anticorrelated pairs indicates a robust relationship between leadership asymmetry and experienced agency. This convergence between behavioral measures of leadership and subjective reports of agency indicates that control and prediction were asymmetrically distributed within dyads.

When considered alongside the intentional binding results, these asymmetries help explain why temporal binding did not emerge uniformly across partners in joint-action conditions. Partners who occupied leader roles and reported stronger agency also exhibited stronger binding effects, whereas followers showed reduced binding. This pattern suggests that temporal binding in joint action depends on how predictive control over the shared outcome is distributed within the dyad.

From this perspective, inter-participant variability in binding under joint action conditions reflects the emergence of asymmetric leader-follower dynamics within an otherwise symmetrical task. Leaders who initiate actions or commit to them more forcefully maintain stronger predictive coupling between their own actions and the shared outcome, which may be associated with enhanced binding and stronger agency attribution. Followers, by contrast, may rely more on reactive coordination, leading to weaker predictive control and, consequently, reduced binding. These results align with models that treat agency as graded and dynamically shared, depending on each partner's relative contribution to the joint action (Wen et al., 2015; Pfeiffer et al., 2013), rather than the emergence of a unified we-agency. This supports recent critiques of the explanatory power of the we-agency concept (Le Besnerais et al., 2024).

The absence of a comparable pattern in the **WC** condition further supports this view. Here, the computer partner deterministically led the interaction, effectively removing the possibility of shared predictive control. Consequently, participants reported little sense of intentional contribution, but the estimated data indicated no difference between the **WC** and **I** conditions in terms of temporal binding. This dissociation suggests that it is not the socialness of the partner, but rather the predictability of the timing of the causal action itself that determines the emergence of agency-related binding effects. Although the computer-controlled partner is overbearing and removes much of the subject's agency over the action, its movements are also smooth and regular. As such, the timing of the button press is easily predictable even though the subject does not exert control over the action. This supports other recent study indicating that predictability is also the key factor in temporal binding effects in tasks with joint action initiation (Le Besnerais et al., 2025). Our design contrasts with the human-robot interaction used by Grynszpan et al. (2019), in which the robotic partner could either initiate movement or flexibly follow the human participant, yielding control when sufficient to counterforce. Such flexibility may have introduced greater temporal uncertainty in the joint action dynamics, which is compatible with the observation that interaction with another human yielded a higher temporal binding effect than interaction with a machine in that study.

## 5 Conclusion

Across individual and joint-action contexts, temporal binding was largely consistent, regardless of whether the partner was human or computer-controlled. This challenges prior assumptions that intentionality or social context is necessary for binding to occur and instead supports the interpretation that binding primarily reflects predictability within the sensorimotor loop. When the causal and temporal relationship between action and outcome is stable, temporal estimates remain largely consistent whether or not agency is constrained by or delegated to a non-human system.

Simultaneously, the relationship between reported sense of agency and binding within human-human dyads revealed a subtler picture. Participants who reported stronger agency in joint actions also exhibited stronger binding, and these differences aligned closely with emergent leader-follower roles in the movement dynamics. Those who took a leader role, i.e., tended to initiate and guide the joint action, reported greater agency and demonstrated enhanced IB effects. Conversely, those in a follower role showed weaker binding and lower subjective agency. Taken together, these patterns suggest that binding is not simply a measure of intention, but a reflection of one's degree of influence within a shared control hierarchy.

The absence of comparable effects in the human-computer joint actions reinforces this interpretation. The computer partner's dominance removed opportunities for shared or alternating control, leading to uniformly low agency reports and no measurable relationship between leadership, SoA, or binding. Yet binding scores were consistent across human-computer joint actions and individual actions, indicating that predictability and regularity can sustain consistent temporal estimation effects even when there is no sense of agency over action initiation and outcome timing. However, we note that participants received only sparse instructions on resolving conflicting movements during joint actions. Therefore, it is also plausible that the participants may nevertheless have acted intentionally in the **WC** condition by following the computer partner's movements. Such “intentional following,” is compatible with reduced experienced authorship over action initiation and outcome timing, as indicated by the consistently low sense of agency reports in this condition. Future research should explicitly dissociate intentional following from unintentional leading to examine how these distinct forms of intentional engagement contribute to temporal binding.

Nevertheless, the dissociation between predictability and sense of agency illustrates a limitation of IB as a diagnostic measure for human-computer joint actions. From an applied perspective, these findings highlight why intentional binding alone cannot serve as a sufficient indicator of “human-like” agency. A system could elicit strong binding simply by maintaining consistent sensorimotor contingencies, regardless of whether the human experiences genuine control or joint intentionality. However, when combined with measures of coordination dynamics and subjective agency alignment, binding may still serve as a sensitive marker of how predictive control is distributed between partners.

Ultimately, we find that temporal binding primarily reflects individual sensorimotor predictions, even when control is distributed across an interacting dyad. This reframes the role of IB in joint action research and human-AI interaction: it may not be a measure of collective “we-agency” *per se*, but may be useful as a quantifiable signature of how effectively partners co-regulate each other's actions.

## Data Availability

The raw data supporting the conclusions of this article will be made available by the authors, without undue reservation.

## Appendix: Questionnaire for participants

Participants were given a simple questionnaire at the conclusion of the session. There were six statements, each responded to on a 7-point Likert scale, with options ranging from “Strongly disagree” (–3) through “Unsure” (0) to “Strongly agree” (+3). The statements were as follows:

1. I could easily tell the difference between trials in which I controlled the pointer myself and those in which the computer also controlled it with me.
2. I could easily tell the difference between trials in which I controlled the pointer myself and those in which the other person controlled it jointly with me.
3. I could easily tell the difference between trials in which the computer and I jointly controlled the pointer and those in which the other person and I jointly controlled it.
4. In trials where I controlled the pointer on my own, I felt like I was intentionally pressing the button.
5. In trials where the computer was controlling the pointer jointly with me, I felt like I was intentionally pressing the button.
6. In trials where the other person was controlling the pointer jointly with me, I felt like I was intentionally pressing the button.

Statements 1, 2, and 3 were primarily given to ensure that the participants' experience broadly aligned with the expectation that each action condition would be clearly distinct. All participants responded positively to statements 1 and 3. Two participants responded negatively to statement 2, and two responded neutrally. Means and ranges for each of these statements were: Q1: mean = 2.67, range = [1, 3]; Q2: mean = 2.02, range = [–1, 3]; Q3: mean = 2.35, range = [1,3]. This indicates that the presence of the computer-controlled partner was clear to all participants, and most participants could detect a human-controlled partner compared to acting independently. Notably, participants were instructed at the start of each block about the type of control condition in effect. Responses to statements 4, 5, and 6 are discussed in the main text.
